# Do inter-hospital comparisons of in-hospital, acute myocardial infarction case-fatality rates serve the purpose of fostering quality improvement? An evaluative study

**DOI:** 10.1186/1472-6963-10-334

**Published:** 2010-12-08

**Authors:** Willem Aelvoet, Nathalie Terryn, Geert Molenberghs, Guy De Backer, Christiaan Vrints, Marc van Sprundel

**Affiliations:** 1 Federal Service of Health, Food Chain Safety and Environment, Brussels, Belgium. Federal Service of Health, Food Chain Safety and Environment; Directorate-General for the Organisation of Health Care Establishments; Datamanagement, Studies; Eurostation Bloc II - first floor - Place Victor Horta 40 bte 10; B-1060 Brussels; 2 Epidemiologie en Sociale Geneeskunde, Universiteit Antwerpen, Antwerp, Belgium; 3 Faculteit Geneeskunde en Farmacie, Vrije Universiteit Brussels, Brussels, Belgium; 4 Interuniversity Institute for Biostatistics and statistical Bioinformatics, Universiteit Hasselt and Katholieke Universiteit Leuven, Belgium; 5 Dept of Public Health, Ghent University, Ghent, Belgium; 6 Division of Cardiology, Ghent University Hospital, Ghent, Belgium; 7 Division of Cardiology, Antwerp University Hospital, Belgium

## Abstract

**Background:**

In-hospital case-fatality rates in patients, admitted for acute myocardial infarction (AMI-CFRs), are internationally used as a quality indicator. Attempting to encourage the hospitals to assume responsibility, the Belgian Ministry of Health decided to stimulate initiatives of quality improvement by means of a limited set of indicators, among which AMI-CFR, to be routinely analyzed. In this study we aimed, by determining the existence of inter-hospital differences in AMI-CFR, (1) to evaluate to which extent Belgian discharge records allow the assessment of quality of care in the field of AMI, and (2) to identify starting points for quality improvement.

**Methods:**

Hospital discharge records from all the Belgian short-term general hospitals in the period 2002-2005. The study population (N = 46,287) included patients aged 18 years and older, hospitalized for AMI. No unique patient identifier being present, we tried to track transferred patients. We assessed data quality through a comparison of MCD with data from two registers for acute coronary events and through transfer and sensitivity analyses. We compared AMI-CFRs across hospitals, using multivariable logistic regression models. In the main model hospitals, Charlson's co-morbidity index, age, gender and shock constituted the covariates. We carried out two types of analyses: a first one wherein transferred-out cases were excluded, to avoid double counting of patients when computing rates, and a second one with exclusion of all transferred cases, to allow the study of patients admitted into, treated in and discharged from the same hospital.

**Results:**

We identified problems regarding both the CFR's numerator and denominator.

Sensitivity analyses revealed differential coding and/or case management practices. In the model with exclusion of transfer-out cases, the main determinants of AMI-CFR were cardiogenic shock (OR_adj _23.0; 95% CI [20.9;25.2]), and five-year age groups OR_adj _1.23; 95% CI [1.11;1.36]). Sizable inter-hospital and inter-type of hospital differences {(OR_comunity vs tertiary hospitals_1.36; 95% CI [1.34;1.39]) and (OR_intermediary vs tertiary hospitals_1.36; 95% CI [1.34;1.39])}, and nonconformities to guidelines for treatment were observed.

**Conclusions:**

Despite established data quality shortcomings, the magnitude of the observed differences and the nonconformities constitute leads to quality improvement. However, to measure progress, ways to improve and routinely monitor data quality should be developed.

## Background

Acute Myocardial Infarction (AMI) is a health problem of paramount importance in terms of frequency, seriousness, social and economic costs, amenability to medical intervention and priority-ranking by policy makers and the community [1,2]. Although guidelines that effectively reduce AMI in-hospital case-fatality rates (AMI-CFRs) exist, they are not uniformly applied [3-7]. Conversely, reports of AMI-CFRs were thought to favorably affect quality initiatives [8]. The relationship between better care and lower AMI-CFRs led several countries, national and international agencies, and consumer's organizations to select AMI-CFR as a quality indicator and to publish individual providers' rates [3,9-11].

To organize the care of AMI patients in Belgium, a three-level structure has been set up. It consists of community hospitals (labeled A) having no catheterization facility, intermediary hospitals (labeled B1) providing coronary angiography but no Percutaneous Coronary Intervention (PCI), and tertiary hospitals (labeled B2-B3) offering PCI and/or Coronary Artery Bypass Graft. A recent study, however, showed unable to establish better outcomes for patients treated in services with catheterization facilities [12].

Current professional knowledge at the time of our study (2002-5) included the need to distinguish between ST-segment elevation myocardial infarction (STEMI) and non-ST-segment elevation myocardial infarction (NSTEMI), because of their different prognosis; the need for reperfusion as quick as possible, by PCI where possible or by thrombolysis otherwise; and, the safety of transport of patients from community to tertiary hospitals [13-15].

Changes in definition and the diversity of the various cardiac troponin assays may have heavily affected AMI incidence rates and AMI-CFRs [16-20].

At the time the study data were registered and coded by the hospitals, the International Classification of Diseases, Ninth Revision, Clinical Modification (ICD-9-CM) did not distinguish between STEMI and NSTEMI, but as from October 2005 new ICD-9-CM regulations provide guidance to do so [21]. Herein STEMI is coded as 410.0-6, 410.8; NSTEMI as 410.7; and AMI "Not Otherwise Specified" (AMI-NOS) as 410.9. It became then possible for one of the authors (NT) to recode a posteriori the data in terms of STEMI or NSTEMI.

According to the OECD "Case-fatality rates measure the proportion of patients with a given diagnosis, here acute myocardial infarction (AMI), who die within a specified time period, here 30 days [11]. Ideally, the case-fatality rates would be based on each individual patient who would be tracked for at least 30 days. However, as most countries do not have unique patient identifiers and lack the ability to track patients after hospital discharge, the indicator is based on unique hospital admissions and restricted to mortality within the hospital. Thus, differences in practices in discharging and transferring patients may influence the findings [11]". Due to the specifics of our administrative data and in alignment with the Agency for Healthcare Research and Quality (AHRQ) definition of the Acute Myocardial Infarction Mortality Rate, our definition of AMI-CFR is exclusively based on hospitalized cases and fatalities within the hospital regardless of any time constraint [9,22].

A fair inter-hospital comparison requires high-quality data [23,24]. Indeed, they have to be sufficiently detailed to allow fair comparison between a hospitals' practices and the then professional knowledge but also to assess the hospitals' organizational performance, especially its transfer policy and the symptom-onset-to-balloon time [25]. Risk-adjustment constitutes a constant threat to the fairness of the comparisons and widely used proprietary risk-adjustment systems may lead to erroneous conclusions [26,27].

Accountability of caregivers and health authorities to the community is internationally considered of paramount importance [28]. In spite of known limitations, public reporting of comparative information about the quality of health care, often derived from administrative data, is frequently put forward as an important quality improvement tool, which attempts to stimulate caregivers to grade up the provision of services and to reassure patients by demonstrating accountability [29-31]. In this context, ensuring data quality is a continuous challenge especially if the same data are used for reimbursement and for measuring quality [31,32].

In an effort to encourage the hospital system to assume responsibility, the Belgian Ministry of Public Health decided to stimulate initiatives of quality improvement. Hereto a limited set of indicators was selected from the AHRQ Inpatient Quality Indicators, including the AMI-CFR [33]. These indicators were to be routinely analyzed, using routinely collected, Belgian hospital discharge records that are known to differ across institutions in quality of data [34] and recognizing that the above-mentioned differences in practices in discharging and transferring patients may influence the findings.

In this study we explore the AMI-CFRs' potential as a quality-improving tool. More precisely we aimed, by determining the existence of inter-hospital differences in AMI-CFR, (1) to evaluate to which extent Belgian discharge records allow the assessment of quality of care in the field of AMI, and (2) to identify starting points for quality improvement.

## Methods

### Data sources

Belgian hospitals are required to register discharge data on each sojourn, which are processed and stored in a dataset called the Minimal Clinical Data (MCD). The MCD contains patient data (among which year of birth, gender, residence, and anonymous hospital and patient identifiers) and stay data (among which year, month and day of the week but, due to privacy restrictions, not the precise date of admission and discharge; length of stay; transfer to another hospital with specification of the type of hospital). It further includes an unbounded number of ICD-9-CM coded diagnoses and procedures but neither results of laboratory investigations, such as cardiac enzymes, nor of technical examinations such as electrocardiograms. Due to privacy regulations a unique identifier, allowing for follow-up of a transferred patient, is lacking. Nonetheless, these data allow for the identification of a cardiogenic shock, the most feared complication of AMI, as well as for the computation of the Charlson co-morbidity index (CCI) (see additional file 1: Charlson), particularly well suited to study AMI-CFRs [2,20,35-37].

Incompleteness and inaccuracy are well-known drawbacks of administrative data; therefore, we compared the MCD data with those from the Ghent and Bruges registries for acute coronary events, which are collected according to the protocol that was originally developed in the WHO Multinational **Moni**toring of Trends and Determinants in **Ca**rdiovascular Diseases (MONICA) project [16,38] (see additional file 2: MONICA definitions). In this project the record form is intentionally kept as simple as possible [38]. It consists of (1) items characterizing the person (date of registration, gender, date of birth and data of onset of the acute attack) and of (2) medical and diagnostic data (whether it was an hospital case or managed elsewhere; whether it was a first or a recurrent event; whether the patient survived at 28 days or not; determination of the diagnostic category based on symptoms, electrocardiograms, serum enzymes and necropsy findings). In addition, the registries of Ghent (covering the city of Ghent (about 250,000 inhabitants)) and Bruges (covering the district of Bruges (about 260,000 inhabitants)) record whether or not a revascularization procedure (PTCA or thrombolysis) was carried out. Coding of all items is checked by both external and internal quality control [38].

In the light of these uniform registration practices and the quality control carried out, we consider the MONICA registries the standard against which we evaluated the MCD.

For the years 2002-2004 and for the age groups between 25 and 74 years, we compared the MONICA hospitalized fatalities (fatal definite (F1) + fatal possible (F2)), the numerator of the AMI-CFR, and MONICA hospitalized cases (non-fatal "definite" (NFl) + "possible" (NF2) + fatal "definite" (F1) + "possible" (F2)), the denominator of the AMI-CFR, with respectively the AMI fatalities and cases in the same age groups of the MCD. Unlike the AHRQ definition, a case fatality in MONICA is defined as death within 28 days after the occurrence of the first symptoms [38]. In the framework of routine analyses of the MCD, for reasons of monitoring over the years, and to comply with the OECD requirements, we preferred to stay in line with the AHRQ model, adopted by the OECD, recognizing the difference in definition of in hospital AMI-CFR between MCD and MONICA. However in Belgium, this difference leads to the very small 0.5% difference in AMI-CFR [39].

Due to privacy regulations and according to the agreement with the hospitals not to perform any analysis on hospital level, we were unable to compare both databases neither on a hospital nor on an individual level. However, to formally assess the completeness of the data we intended to compare on register level the number of cases as well as the case-fatality rates of the Ghent and Bruges registries with the corresponding MCD. To do so log-linear models were to be fitted with the number of cases as the dependent variable, and gender, place and registry as covariates. The need to include registry as a covariate was to be examined by leaving registry out from the explanatory variables. Fitting log-binomial regression models [40] would allow us to observe a possible difference in risk ratio between both registries and MCD, implying either an underestimation or an overestimation of case-fatality rates by the MCD. Notice that, according to the MONICA protocol, fatalities occurring within the first hour after hospitalization were recorded by the registries of Ghent and Bruges.

### Definition of the study population

Building on the work of the Agency for Healthcare Research and Quality (AHRQ) [9] we selected, for the years 2002-2005, from the MCD all stays having AMI as principal diagnosis (ICD-9-CM code 410.*1, the fifth digit indicating an initial episode of care). Cases with no information regarding vital status at discharge or aged less than 18 years or, to avoid double counting of patients when computing rates, transferring-out to another short-term hospital were excluded. We also excluded short-term hospitals registering less than 20 cases a year. Applying these criteria we obtained our study population consisting of 46,287 cases (30,271 males and 16,016 females) and 7,099 fatalities (3,841 males and 3,285 females), registered in 109 short-term hospitals.

### Design

Our aim was to identify, at the same time, hospitals with higher quality of care, for benchmark and exemplary function purposes, and hospitals with lower quality, to help them improve their performance. To this effect, the AMI-CFR of each individual hospital was compared with the corresponding rate of the whole of the other Belgian hospitals.

To assess hospital-specific rates of AMI-CFR a cohort study of all hospitalized AMI cases of the years 2002 to 2005 was carried out. Hereby, two types of analyses were performed: a global one focusing on the AMI-CFRs of the entire time span of the study (the "period") and a temporal one (the "trend"), focusing on its per-semester evolution and intended to refine the initial, global assessment by taking temporal evolution into account.

It has been suggested that in analyses, founded on administrative databases, confounding effects of unmeasured or mismeasured variables can be equivalent in magnitude to the effect of the association of interest and cannot be ruled out as an explanation of rather small, yet statistically significant effect sizes, such as a relative risk (RR) of, say, 0.75 to 1.35, which are roughly each other's inverses [41]. To take this caution against over-interpretation into account, we defined an inconclusive zone, where the AMI-CF rate or AMI-CFR trend of a hospital, compared with these of the other hospitals, should not be interpreted as being "higher" or "lower," but rather be considered "inconclusive." First, we computed a hospital's relative risk (RR) of having a higher/lower period or trend AMI-CFR than those of the other hospitals. Subsequently, this RR allowed us to calculate a "departure," equaling (RR-1) × 100, which we used to define an "inconclusive zone." Regarding the period, we fixed the lower and upper boundaries of the inconclusive zone at a departure of - 25% and of + 35%, respectively, in line with the aforementioned relative risks. In the absence of literature regarding important departures of the trend, we arbitrarily fixed these boundaries at - 5% and + 5%, allowing for a maximal increase of + 41% or for a maximal decrease of -30%, respectively, of the AMI-CFR during the entire time span of the study. The technical details of our approach are described in the paragraph devoted to the statistical analysis.

The results of the analyses regarding hospitals outside the inconclusive zone, were interpreted according to the degree of statistical evidence. We labeled this evidence as: 1) "strong," if the probability of finding a departure, as important or bigger than that of the hospital under consideration, was smaller than or equal to 0.05 divided by the number of hospitals to be compared (the so-called Bonferroni correction for multiple comparisons [42]); 2) "moderate," if this probability was smaller than or equal to 0.05 but greater than 0.05 divided by the number of hospitals to be compared; 3) "weak," otherwise [34]. For both the period and the trend analyses, two AMI-CFRs were computed: a first one in which transferring-out cases are excluded, so as not to compute national rates with duplicate observations [9], and a second one in which all transferred cases are excluded, to allow for a comparison between hospitals in the subset of cases they treated from uptake to discharge.

We also aimed at identifying transferred patients using composite keys consisting of a patient's year of birth; gender; residence; year and month of admission and discharge; and day of the week of discharge ("discharge" facility's data) or admission ("intake" hospital's data). In these patients, we intended to compare the CCI distributions before and after transfer to evaluate the comparability across hospitals regarding the coding of co-morbidity, a component of the risk-adjustment in our regression analyses. From the viewpoint of quality of care, we also compared in these patients the time span between admission and transfer with the then prevailing guidelines.

### Statistical methods

For our analyses, we used so-called fixed-effects models, motivated by the fact that the entire population of Belgian hospitals is considered, rather than sampling from them. We performed multivariable logistic regressions [43], aiming to identify outlying hospitals, i.e. characterized by an important and statistically significant, Bonferroni-corrected [24] departure from the other hospitals' AMI-CF rate or AMI-CFR evolution over time. Given that we cover the short time span of only eight semesters, we merely fitted models with a linear time trend, which for brevity we called "trend". By incorporating an interaction term in the logistic regression between a linear time trend, expressed in semesters, and individual hospitals, those hospitals with an abnormal evolution in time were identified. Precisely, we compared the slope of each hospital's time trend with that of the other hospitals using linear contrasts.

When the outcome of interest, such as the percentage of fatal cases in the AMI case fatality rate, exceeds 10%, or if the odds ratio is greater than 2.5 or less than 0.5, the estimation of the relative risk by the adjusted odds ratios, derived from the logistic regression no longer adequately approximates the relative risk, which may become heavily biased. Indeed, the more frequently that outcome occurs, the more the odds ratio overestimates the relative risk when it is greater than 1 or underestimates the relative risk when the odds ratio is less than one [44]. To reduce this bias, we used the approximation of the RR by Zhang [44], which has been used to compute the aforementioned departure. The relation between RR_Z _and the odds ratio is given by RR_Z _= OR/((1-P0) + (P0 *OR), where P0 indicates the incidence of the outcome of interest in the non-exposed group [44].

Following the AHRQ model [22], we carried out two types of main analyses: a first one wherein transferred-out cases were excluded and a second one with exclusion of all transferred cases. In each analysis, adjustment was made for five-year age groups, gender, per-semester evolution of the AMI-CFRs, and shock.

To account for correlation within the data, rescaling techniques were used [45]. To study a possible national upward trend we used so-called Generalized Estimating Equations (GEE), a refinement of the logistic regression that corrects for within-hospital, generally within-unit, correlation [46].

### Feedbacks

The results of the analysis serves two purposes: feedback to the hospitals to enable improvement of care, and feedback to the Belgian College of Physicians to identify hospitals with "strong" evidence of either superior or inferior quality.

The feedback to the hospitals consists mainly of (1) a graphical display of the "departure" of each of the hospitals from the rate and trend of the other hospitals, and (2) an anonymous and tabular representation of these departures as well as of an indication of the level of statistical evidence. An aid in the interpretation, combining the information of both the period and trend analyses, is provided alongside. Its decision tree is given in the Appendix of the Supplementary Materials (see additional file 3: decision tree).

In the feedback to the Belgian College of Physicians, we present an "Average" and two "Outlying" categories of hospitals. For the period analysis, a first, outlying category, the 'high AMI-CFR' group, consists of those hospitals with a departure of >+35% and statistically significant (Bonferroni-corrected). A second, outlying category, the 'low AMI-CFR' group, consists of those hospitals with a departure of <-25% and statistically significant (Bonferroni-corrected). The other hospitals are grouped into the 'average AMI-CFR' group. A similar approach is followed regarding the trend, with boundaries at +5% and -5%.

The decision tree is identical to that, used in the feedback to the hospitals, except that hospitals recommended for an external audit are now divided in "high AMI-CFR" and "low AMI-CFR" groups and that the other hospitals are regrouped in an "average AMI-CFR" group.

### Sensitivity analyses

Due to the prognostic differences, related to the type of AMI, its complications and treatment modalities, several sensitivity analyses were carried out. After an initial analysis, modeling AMI-CFR adjusted for age, gender, CCI and time (period as well as trend), subsequent analyses were carried out, consecutively introducing a variable mentioning whether shock was present at admission or not, and a variable indicating whether or not an angioplasty had been performed. We repeated the subsequent analyses, this time replacing shock by a variable describing the type of AMI i.e. STEMI, NSTEMI or AMI NOS. We performed these analyses twice: firstly after exclusion of the transferred-out cases and subsequently after exclusion of all transferred cases. In the trend analyses, we additionally modeled an interaction term between trend and individual hospitals. To summarize the effect of including additional explanatory variables into the model, we classified for each model the hospitals into seven categories, ranging from strong, then moderate, to weak evidence of finding themselves above or below the inconclusive zone.

A last category consisted of hospitals showing no interpretable departure.

In so doing, we were able to compare between the hospitals their changes of category due to the modeling process. Because differences in hospitals' discharge policy may influence their AMI-CFRs we also compared the hospitals' cumulative AMI-CFR at five points in time, namely at the 7th, 14th, 21st, and 28th day of hospitalization, and at the latest day of discharge of any AMI-case [11]. Given the changing numerators of the rate over time, we based our comparison on the hospitals' change in deciles of AMI-CFR. To this end, we computed a maximum change in relation to the initial AMI-CFR. We further determined for each hospital the maximum length of stay (LOS) of any patient whether deceased or not, and the maximum LOS of any deceased patient, both after exclusion of the transfer-out cases and after exclusion of all transferred cases.

To provide an idea of the variability of some major covariates we determined across hospitals and by level of care, averages of the lower quartile, mean, median and upper quartile of the CCI, LOS and rate of shock. In the same way we computed the average 95th CCI-percentile and the ranges of the LOS.

The study being (1) of a retrospective, non-interventional type and (2) anonymous with respect to both hospitals and patients, no approval by an ethics committee is required under the Belgian law.

## Results

### Patient and hospital characteristics by level of care

The MCD dataset consisted of 46,287 cases and 7,099 fatalities [AMI-CFR: 15.34 (95% CI: 15.01;15.67) from 109 hospitals. The majority of cases (65,4%) and of the fatalities (54.1%) were males. In females the AMI-CFR was higher than in males [age-adjusted OR: 1.13 (95% CI: 1.04; 1.22)]. In tertiary hospitals, younger age groups (Pearson chi-square with 10 df = 794.7; p < 0.0001), males (Pearson chi-square with 2 df = 214.5; p < 0.0001) and shock (Pearson chi-square with 2 df = 6.29; p = 0.043) are over-represented (Table 1).

**Table 1:** Patient and hospital characteristics by level of care

	Level of care								
	Type A			Type B1			Type B2-B3		
Patient charateristics									
Age	N	%	CFR	N	%	CFR	N	%	CFR
<40 y	217	1.5	2.8	133	1.9	3.8	508	2.1	4.7
40-59 y	988	6.7	5.6	656	9.1	3.8	2,331	9.6	4.3
50-59 y	2,047	13.9	6.4	1,226	17.1	6.0	4,721	19.4	4.7
60-69 y	2,643	18.0	10.2	1,242	17.3	10.1	5,13	21.0	8.7
70-79 y	4,165	28.3	19.0	1,971	27.4	16.2	6,705	27.5	15.4
80 y+	4,641	31.6	30.5	1,959	27.3	30.6	5,004	20.5	29.2
**Gender**									
Males	9,095	61.9	15.2	4,475	62.3	12.7	16,708	68.5	11.3
Females	5,606	38.1	22.9	2,712	37.7	21.4	7,691	31.5	18.1
Total	14,701		18.1	7,187		16.0	24,399		13.5
**Shock**									
Present	1,313	8.9	75.5	611	8.5	80.2	2,294	9.4	68.2
Absent	13,388	91.1	12.5	6575	91.5	10.0	22105	90.6	7.8
**Type of AMI**									
STEMI	1,497	63.0	16.18	775	73.1	14.76	2,036	67.3	12.39
NSTEMI	251	14.2	7.48	88	9.5	7.03	366	13.0	7.61
AMI NOS	917	22.8	43.9	285	17.4	41.67	884	19.7	27.97
									
**Hospital characteristics**									
**Number**	61			19			29		
**Volume 2002-5**, **transfers out excluded**									
Minimum	95			239			312		
Lower quartile	149			277			584		
Median	194			355			776		
Mean	241			378			841		
Upper quartile	309			487			1122		
Maximum	817			660			1596		
**Volume 2002-5**, **all transfers excluded**									
Minimum	69			133			292		
Lower quartile	123			200			484		
Median	174			265			685		
Mean	201			281			708		
Upper quartile	249			332			801		
Maximum	798			542			1448		

Patient characteristics:

N: number of cases; %: column percentage; CFR: AMI-CFR in %

STEMI: ST-segment elevation myocardial infarction; NSTEMI: non-ST-segment elevation myocardial infarction; AMI NOS: AMI not otherwise specified.

Level of care: community hospitals (labeled A) having no catheterization facility, intermediary hospitals (labeled B1) providing coronary angiography but no Percutaneous Coronary Intervention (PCI), and tertiary hospitals (labeled B2-B3) offering PCI and/or Coronary Artery Bypass Graft.

Hospital characteristics:

Minimum, Maximum: minimum and maximum number of observations per type of hospital

Lower quartile, Median, Mean, Upper quartile: percentiles of the distribution of the number of observations per type of hospital

The age-adjusted mortality was higher in Type A [OR:1.17; 95%CI(1.09;1.26)] hospitals than in Type B2-B3 hospitals. However, we were unable to establish a significant difference in age-adjusted mortality between Type A en Type B1, and between Type B1 and Type B2-B3 hospitals (Table 1). Males had lower mortality figures than females [OR:0.58; 95%CI(0.55;0.61)]. The sex-adjusted mortality was higher in Type A [OR:1.37; 95%CI(1.30;1.45)].and Type B1[OR:1.18; 95%CI(1.10;1.27)] hospitals than in Type B2-B3 hospitals. Type A hospitals also displayed a higher sex-adjusted odds ratio [OR:1.16; 95%CI(1.08;1.26)] than Type B1 (Table 1).

Type B2-B3 hospitals had a lower CFRs in case of cardiogenic shock {(OR_A_vs_B2-B3_:1.14 [95%CI(1.23;1.67)] and OR_B1_vs_B2-B3_:1.89 [95%CI(1.51;2.21)])}, and Type A hospitals also had a lower CFR than Type B1 (OR_A_vs_B1_:0.76 [95%CI(0.60;0.96)]). Type B2-B3 hospitals had also a lower CFRs in case of absence of cardiogenic shock {OR_A_vs_B2-B3_:1.70 [95%CI(1.58;1.82)] and OR_B1_vs_B2-B3_:1.32 [95%CI(1.20;1.45)] }, but now Type A hospitals had a higher CFR than Type B1 (OR_A_vs_B1_: 1.29 [95%CI(1.17;1.41)]) (Table 1).

With respect to STEMI cases we observed higher AMI-CFRs of Type A and B1 versus Type B2-B3 hospitals {(OR_A_vs_B2-B3_:1.36 [95%CI (1.27;1.47)] and OR_B1_vs_B2-B3_:1.22 [95%CI (1.12;1.34)])} and of Type A versus Type B1 hospitals (OR_A_vs_B1_: 1.11 [95%CI (1.01;1.22)]). In NSTEMI cases we observed substantially lower AMI-CFRs in Type B2-B3 hospitals {(OR_A_vs_B2-B3_:2.01[95%CI (1.79;2.26)] and OR_B1_vs_B2-B3_:1.84 [95%CI (1.55;2.18)])} (Table 1). In contrast we were unable to demonstrate either a difference in AMI-CFR between Type A and Type B1 hospitals or evidence of any deviating AMI-CFR in cases of AMI-NOS. The proportions of cardiogenic shock were very high, viz 9.9% in STEMI, 4.2% in non-STEMI infarctions and even 12.7% in the AMI NOS group.

On the hospital level we noticed huge variability across institutions of the same type regarding CCI, length of stay and to a lesser extent shock (expressed in %). However the distribution of these variables was very similar in the three types of hospitals (data not shown).

We also observe a huge variability in volume, i.e the number of cases, within and between types of hospital (Table 1). Based on the volumes of the lower quartile, the median and the upper quartile of the Type B1 hospitals in the data, transfers out excluded, we grouped the hospitals in four classes. We observed a huge variability in volume between and within Types of hospital (Pearson chi-square of 300019 with 6 df;p < 0.0001) with excesses of low volume hospitals in Type A hospitals (41 out of the 61 of these hospitals found themselves in the lowest class) and of high volumes in Type B2-B3 hospitals (26 out of the 29 of these hospitals found themselves in the highest class). The Type B1 hospitals were almost equally distributed over the four classes. Similar findings were encountered in the analysis on the data with exclusion of all transfers.

### Comparison between MCD and MONICA

The comparison of the MCD with MONICA (Ghent and Bruges) shows important differences between both datasets (Table 2), characterized by more fatalities and significant higher case-fatality rates in the MONICA registry. Notice that in the MONICA registry 54 out of the 190 fatal cases occurred within the first hour after hospitalization. At that moment these patients probably found themselves in the emergency services, where in Belgium as yet no specific ICD codes are systematically registered. Regarding the number of cases, we were unable to determine a significant difference between both datasets, the difference in the Pearson chi-squared statistics of our log-linear models being not significant (p = 0.19). Conversely, we observed a significant difference in risk ratio implying an important underestimation of case-fatality rates by the MCD (RR:0.39[95%CI:0.31;0.51]). However in the absence of a significant interaction term between registry and place (p = 0.82) we were unable to establish a differential underestimation according to place. Leaving out the fatalities occurring within one hour after admission led to similar results.

**Table 2:** Comparison MCD and MONICA (districts of Bruges and Ghent); 2002-4.

Bruges 2002-2004							Ghent 2002-2004					
	Females						Females					
	Fatal			Total			Fatal			Total		
Age	MONICA			MCD	MONICA	MCD	MONICA			MCD	MONICA	MCD
	<1 h°	≥1 h°	Subt.°°				<1 h	≥1 h	**Subt**.			
25 - 34 y	0	0	0	0	0	1	0	0	0	0	2	3
35 - 44 y	0	0	0	0	4	6	0	0	0	0	3	2
45 - 54 y	0	1	1	1	12	10	1	2	3	2	18	13
54 - 64 y	0	6	6	1	23	23	5	4	9	2	29	28
65 - 74 y	5	14	19	6	71	65	6	11	17	5	59	54
Total	5	21	26	8	110	105	12	17	29	9	111	100
	**Males**						**Males**					
	Fatal			Total			Fatal			Total		
Age	MONICA			MCD	MONICA	MCD	MONICA			MCD	MONICA	MCD
	<1 h	≥1 h	Subt.				<1 h	≥1 h	Subt.			
25 - 34 y	0	0	0	0	1	1	0	0	0	0	4	3
35 - 44 y	0	0	0	0	23	20	0	2	2	1	34	29
45 - 54 y	2	7	9	3	104	89	4	3	7	4	87	95
54 - 64 y	1	10	11	4	117	114	8	22	30	11	112	109
65 - 74 y	9	31	40	12	162	141	13	23	36	19	137	138
Total	12	48	60	19	407	365	25	50	75	35	374	374

°: </≥1 hour: number of fatal cases having sojourned less than/at least one hour at the hospital before dying. This item is only registered in the MONICA registry; the MCD merely provides the day of decease without indicating whether the event took place within the first hour of admission or later on in the hospital stay.

°°:Subt.: subtotal (MONICA registry): number of fatal cases having sojourned at least one hour at the hospital before dying and of the number of fatal cases having sojourned less than one hour at the hospital before dying

MCD: Minimal Clinical Data; MONICA: Monitoring of Trends and Determinants in Cardiovascular Diseases register

Notice that the number of (mortality) cases registered in the MCD alternately was larger or smaller than those of MONICA.

We were also able to compare PTCA figures between MONICA and MCD. For the years 2002-4 MONICA-Ghent registered 83 PTCAs in males and 19 in females versus respectively 102 and 20 PTCAs in the MCD. For Bruges these numbers amounted to 87 and 20 in MONICA versus 149 and 30 in the MCD. We paired the data and computed their proportions as well as the corresponding 95% confidence interval [47]. Apart from the numbers for females in Ghent, the MCD numbers of PTCA exceeded significantly (the lower bound of the CI being ≥1) those of MONICA.

For the same years we also compared the recurrent events registered in MONICA with the AMI cases in the MCD mentioning in addition an "old myocardial infarction" (ICD-9-CM code: 412) as a secondary diagnosis. This exercise also showed substantial differences between MONICA and MCD. In Ghent MONICA totaled 53 recurrent events in males and 11 in females, versus 22 and 3 respectively in the MCD. For Bruges these numbers amounted to 76 and 14 in MONICA versus 49 and 6 in Bruges. Each time the MONICA figures significantly outnumbered (the lower bound of the CI being ≥1) those of the MCD.

### Transfers

From the MCD data, before the exclusion of transfer cases, we identified 6,555 stays mentioning only a transfer-in from another hospital, 12,409 stays mentioning only a transfer-out to another hospital, and 4592 mentioning both a transfer-in and a transfer-out. We only succeeded to pair respectively 2,524, 4592 and 2657 transfers.

In the subset of 2,524 paired transfer-in stays, we also assessed the accuracy of our co-morbidity data. The first quartile, median and third quartile of the distribution of the CCI amounted to 0, 0 and 2 in the "discharging" and to 0,1 and 2 in the "intake" facilities respectively. We also determined the time of referral, which in over 50% of the cases exceeded 24 hours.

### Sensitivity analyses

In 27 hospitals neither the adjustments used nor the stays excluded according to type of transfer, altered the category of evidence to which they belonged. In 45 hospitals the change did not exceed 2 categories; in 31 hospitals the change equaled 3 categories, without crossing the overall rate; and, in 5 tertiary hospitals the change amounted at least to 3 categories and crossed the overall rate. In the latter cases poorer AMI-CFRs were found when adjustment was made for shock or STEMI and for carrying out an angioplasty.

The range of the point estimators of the departures was considerable and varied substantially across the fitted models (Table 3).

**Table 3:** Range of the point estimators of the "departures" according to the fitted model.

		I		I + Sh		I + Sh + A		I + St		I + St + A	
		Max	Min	Max	Min	Max	Min	Max	Min	Max	Min
No transfer-out cases	Period	140	-64	182	-63	152	-70	97	-59	91	-64
	Trend	25	-38	30	-41	29	-42	27	-54	26	-31
All transferred cases excluded	Period	153	-62	196	-65	163	-71	104	-64	84	-63
	Trend	29	-42	39	-47	42	-48	30	-32	31	-33

I: initial model (age, gender and CCI); I + Sh: initial model + shock;

I + Sh + A: initial model + shock + angioplasty; I + St: initial model + STEMI;

I + St + A: initial model + STEMI + angioplasty.

In a first series of analyses, wherein transferred-out cases, were excluded, the cumulative AMI-CFR, adjusted for age, gender, CCI and shock, showed a huge inter-hospital variability in AMI-CFR over time. The maximum change in deciles amounted to 0, 1, 2, 3 and 4 or 5 deciles in 21, 49, 25, 12 and 2 hospitals respectively. Analogously, after exclusion of all transferred cases, a maximum change in deciles of 0, 1, 2, 3, 4 or 5 was observed in 24, 58, 15, 11 and 1 hospitals, respectively.

Similarly, excluding the transferred-out cases, we observed across hospitals ranges of the maximum LOS of any patient from 36 to 337 days and from 12 to 233 days in deceased patients. Very similar ranges from 36 to 243 days and from 12 to 232, respectively, were obtained after exclusion of all transferred cases.

### Determinants and evolution of the national AMI-CFR

Shock (adjusted ORs of 23.0[95%CI: 20.9;25.2] and of 21.0 [95%CI: 19.0;23.2]) proved to be by far the strongest determinant of AMI-CFR in all the models, followed by age (adjusted ORs between 1.35 and 1.37), and severe co-morbidity (for example in the Initial model 1 a CCI of four has an adjusted OR of 1.09^4 ^= 1.41. Indeed, CCI being modeled as an interval variable, an adjusted odds ratio between successive levels of CCI equals 1.09.) (Table 4). The adjusted odds ratios of gender and trend, although statistically significant, found themselves in the inconclusive zone.

**Table 4:** Determinants and evolution of AMI-CFR. Mutually adjusted odds ratios and 95% confidence intervals ().

	Initial 1	Initial 2	Shock 1	Shock 2
Age (5-year groups)	1.37 (1.35; 1.40)	1.36 (1.34; 1.39)	1.36 (1.34; 1.39)	1.35 (1.32; 1.38)
Males	0.90 (0.84; 0.96)	0.92 (0.86; 0.99)	0.91 (0.84; 0.98)	0.93 (0.86; 1.01)
CCI	1.09 (1.08; 1.10)	1.07 (1.06; 1.09)	1.06 (1.05; 1.08)	1.05 (1.04; 1.06)
Trend (semester)	0.98 (0.96; 0.99)	0.98 (0.97; 1.00)	0.98 (0.96; 0.99)	0.99 (0.97; 1.00)
Shock	. . .	. . .	23.0 (20.9; 25.2)	21.0 (19.0; 23.2)
Community vs tertiary	1.21 (1.12; 1.30)	1.47 (1.37; 1.59)	1.39 (1.28; 1.50)	1.61 (1.48; 1.75)
Intermediary vs tertiary	1.08 (0.99; 1.19)	1.38 (1.25; 1.52)	1.23 (1.11; 1.36)	1.48 (1.33; 1.65)

AMI-CFR: Acute myocardial infarction case-fatality rate

Initial 1: odds ratios adjusted for age, gender, CCI, and trend (transfers-out excluded)

Initial 2: odds ratios adjusted for age, gender, CCI, and trend (all transfers excluded)

Shock 1: odds ratios adjusted for age, gender, CCI, trend, and shock (transfers-out excluded)

Shock 2: odds ratios adjusted for age, gender, CCI, trend, and shock (all transfers excluded)

### Inter-hospital comparison

**Figure 1:**
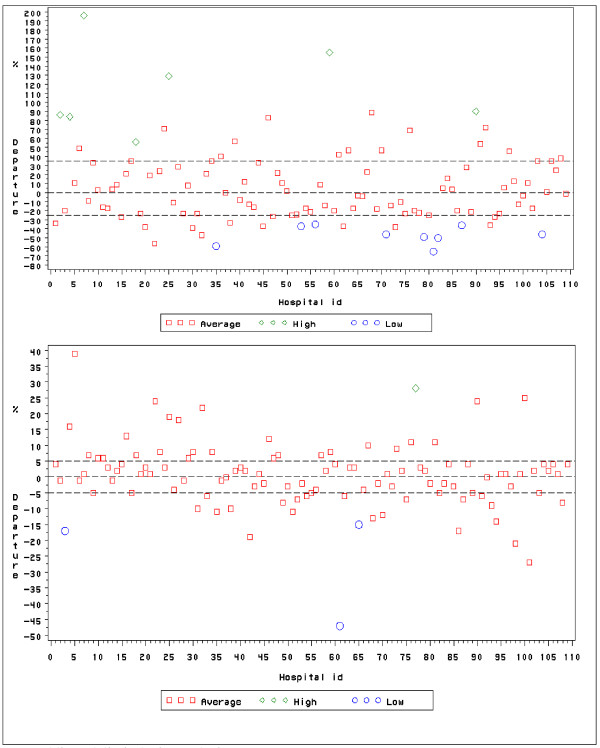
**Inter-hospital differences in AMI-CFR, period (upper part of the figure) and trend (lower part of the figure), all transfers excluded. Belgium 2002-5**. Dotted lines delimit the inconclusive zone. Hospital id: anonymous hospitals' identification number.

We observed substantial and statistically significant inter-hospital differences in AMI-CFRs, both in the period and the temporal analysis, based on a model with age, gender, CCI and shock as explanatory variables. These differences arose in both the models with exclusion of transferred-out cases and those wherein all transferred cases were excluded. From the model wherein all transferred cases were excluded, we represented in Figure 1 the Bonferroni-corrected, outlying hospitals by green diamonds ("high") or blue circles ("low") and the other hospitals by red squares ("average"). Departures ranged from -65% up to +196% in the period, and from -47% up to +39% in the trend analysis, resulting in seven "high AMI-CFR" and nine "low AMI-CFR" outlying hospitals in the period and one "high AMI-CFR" and three "low AMI-CFR" outlying hospital in the trend analysis. For the model with exclusion of transferred-out cases, we observed departures ranging from -60% to +185% in the period and from -45% to +30% in the trend analysis, resulting in four "high AMI-CFR" and eight "low AMI-CFR" outlying hospitals in the period and one "high AMI-CFR" and one "low AMI-CFR" outlying hospital in the trend analysis.

The same analyses, performed on the subset of tertiary level hospitals, also showed marked and statistically significant inter-hospital differences in AMI-CFRs in both models. In the model with transfer-out cases excluded, the departures in the period analysis ranged from - 36% to +70%, resulting in two "high AMI-CFR" and one "low AMI-CFR" outlying hospitals. In the trend analysis no significant departures were observed. In the model with all transfers excluded we observed one "high AMI-CFR" outlying hospital in the period analysis (departure +60%) and one "low AMI-CFR" outlying hospital in the trend analysis (departure - 14%).

According to type of cardiac care provided, we found important, i.e. beyond the inconclusive zone, and statistically significant differences between community and tertiary level hospitals, except for "Initial 1 model" (Table 4) where, in spite of a statistical significant difference, no firm conclusion could be made, the excess being situated in the inconclusive region. The differences between intermediary and tertiary level hospitals fell outside the inconclusive zone for the models wherein all transfers are excluded { model "Initial 2": OR_B1_vs_B2-B3_:1.38 [95%CI (1.25;1.52)]) and model "Shock 2" OR_B1_vs_B2-B3_:1.48 [95%CI (1.33;1.65)])} (Table 4).

The fitted models showed very good discrimination properties reflected by areas under the receiver operating characteristic (ROC) curves between 0.74 and 0.85 [48].

## Discussion and Conclusions

### Data quality of the MCD

Our results suggested numerous quality-of-data-related problems and prompted a very cautious interpretation of the quality-of-care-related findings. At first, AMI is characterized by diagnostic uncertainty, manifesting itself in marked differences in sensitivity and specificity of the measurement of myocardial proteins, ECG recordings and imaging modalities [18,49]. This may be reflected by the denominator differences of the AMI-CFR we found between the MCD and MONICA data. Part of these differences is probably due to the absence of national guidelines leaving the choice of the diagnostic criteria to the individual clinicians, whereas in MONICA univocal diagnostic criteria were used. In a German study [50], similar to ours, the hospital's AMI-CFR amounted to 13.5% versus the 28% derived from the German MONICA data, whereas in ours these rates amounted to 7.5% and 19,0%, respectively.

The comparison between MONICA and the MCD, unfortunately very limited, due to privacy and agreement regulations, shed light on other quality-of-data problems. The outnumbering in PTCA of the MCD may illustrate the need of univocal definitions of AMI and coding practices and may reflect the propensity of administrative data for maximizing coding. By contrast the deficient registering of old myocardial infarctions, which is financially not rewarding in our prospective payment based reimbursement of the hospitals, comes as no surprise.

We also tried to assess as much as possible the quality-of-data through internal comparisons. Thus and contrary to the findings in a recent publication stressing the sensitivity of the CCI to coding practices [27], our comparison of the CCI distribution in transferred patients did not suggest too large before-after transfer differences. However, our sensitivity analyses seemed to indicate a sizable inter-hospital variability in the accuracy of the data.

The completeness of the data also was a cause for concern. Since about 50% of the fatal STEMI cases occur in the first two hours after the onset of the symptoms, the under-registration of fatalities may be due to the lack of ICD-coding of the pathologies during a stay in an emergency department [4]. Important information such as symptom-onset to needle-time, the time lag between the onset of symptoms and the initiation of the treatment, is lacking in our data, as well as pharmacological treatment details, which are essential with respect to quality assessment, especially in primary hospitals. Also, although low socio-economic status (SES) is related with lesser AMI-outcomes, we have no information regarding SES [51].

Further, one has to take the basic tension into account that exists between using the same data for reimbursement of the hospitals and for quality improvement purposes. Indeed, in the former case the purpose is to maximize the coding of complications and co-morbidities (e.g., CCI), while in the latter it is to restrict it to conditions really affecting care [32]. Our shock data may well illustrate this phenomenon. For example, we observed rates of 9.9% and 4,2% in STEMI and non-STEMI infarctions, respectively, which are substantially higher than the 5 to 8.6% and 2.5% in the literature, respectively, and to which, in addition, the 12.73% of the AMI-NOS are to be added [20,37]. The doubling of the reimbursement rate in these cases reflects the possible financial gains through maximization, but the high CFRs in case of cardiogenic shock seem not to indicate important efforts of maximization.

Whereas in Belgium it is impossible to routinely obtain 30-day mortality data of discharged patients, they are very well comparable to the in-hospital mortality data [39].

By contrast, the comparison with MONICA revealed a substantial proportion of probably early fatalities not registered in the MCD. As a result, the in-hospital AMI-CFR may chiefly be biased in the initial phases of the fatal process, preventing the in-hospital AMI-CFR to be used as a reliable estimator of the population AMI-CFR. In that respect population-based, acute coronary syndrome registries remain irreplaceable.

Since we were not able to assess whether the proportion of unrecognized, early AMI deaths is equally distributed across hospitals, the inter-hospital comparison may, apart from unevenly distributed, dubious coding practices [34], be biased as well.

### Statistical modeling

To achieve the fairest possible inter-hospital comparison we performed two types of multivariable logistic regressions leaving out respectively transferred-out cases, and all transferred cases. In both types of analysis a risk-adjustment for age, gender, CCI and cardiogenic shock was performed. All the models had a C-index, a measure of the discriminative performance of the models, of 0.832 to 0.844, that are comparable to those obtained in a study evaluating five risk scores (PURSUIT, GUSTO-1, GRACE, SRI and EMMACE) for risk stratification of acute coronary syndromes [52]. The authors of this study conclude that simpler risk models had comparable performance to the more complex ones [52]. The Simple Risk Index (SRI) for instance, which consists of age, systolic blood pressure and heart rate, is very similar to our here-mentioned model.

In our models, we kept away from inserting either angioplasty - not to model away the effect of angioplasty - or STEMI, because of both its correlation with cardiogenic shock and its difficult-to-handle class of AMI-NOS (about 13% of our study population and a case-fatality rate of 86.2%), prone to biased analyses [53].

To maximally avoid the inclusion of iatrogenic, in-hospital complications into the risk-adjustment, we also preferred the CCI, based exclusively on chronic conditions to widely used discharge abstract-based software, which fail to distinguish co-morbidities from complications [26,27]. The choice of including cardiogenic shock into the risk-adjustment is an arguable point; being much more often a non-iatrogenic phenomenon than a complication, we decided to incorporate it into our risk-adjustment. Indeed a recent article devoted to cardiogenic shock stated that MI with LV failure remains the most common cause of CS but also that approximately three fourths of patients with CS complicating MI develop shock after hospital presentation of which in some, medication use contributes to the development of shock [20].

Hierarchical models, usually taking the form of so-called random-effects models, would have been an alternative to the logistic regression approach we used. However, outlier detection based on such models is currently methodologically underdeveloped. Indeed, the theory dealing with outliers still has to be further developed for linear mixed models, with even less development for non-linear mixed models [46,54]. Finally, in the random-effects models the hospitals in the set of data are considered a random sample from the larger population of all hospitals, contrary to fact in our study wherein the entire population of Belgian hospitals is considered, rather than sampling from them.

### Inter-hospital comparison

Two methods of calculating AMI lethality are included in the AHRQ's Inpatient Quality Indicators. The first one (excluding transferred-out cases) ensures the inclusion of all AMI patients. The second method (excluding all transferred cases) reflected the desire of users to have an alternative method of measuring AMI mortality that excluded patients transferred from another hospital. However, this approach results in the loss of transferred AMI patients from any quality measurement. Therefore, in order to allow both types of interpretation we presented the results obtained from both methods.

While important, the above-mentioned quality-of-data limitations led inescapably to the question whether the MCD were "good enough" to carry out an inter-hospital comparison [55]. Contextual reasons including the accountability of both the hospitals and the authorities, as guarantors of the quality of health care, brought us to do so as well as the conviction "that what cannot be measured cannot be changed" [56]. Also one had to keep in mind that administrative data, such as the MCD, not only are the most accessible comparative data source for examining all patients admitted to a hospital, but also the only ones allowing a nationwide inter-hospital comparison with at least a minimal risk-adjustment [56-58]. Further, the magnitude of the observed inter-hospital differences in AMI-CFRs - the highest AMI-CFR is six times higher than the lowest - and the influence of type of hospital warrant further inquiry. Indeed, the principal interest of our study in this regard rests on its ability of screening substandard or above-standard care, to be completed by a formal assessment, and its stimulating effect on initiatives of quality improvement.

From the transfer exercise for instance we learned that, going against the prevailing guidelines [14], a considerable number of transfers was realized more than 24 hours after intake, suggesting the carrying out of an elective rather than a rescue angioplasty. The case of the five tertiary level hospitals, which in the sensitivity analysis showed the greatest AMI-CFR variability, may be related to this phenomenon. Indeed, it may that these centers frequently carry out this type of angioplasty, as it was precisely the inclusion in the modeling process of shock, underrepresented in this type of patients, and angioplasty that caused the change in AMI-CFR. Since this result appeared during the modeling process, one may assume it not to be an artifact but rather an expression of sub-optimal care.

Another intriguing finding, which requires elucidation, was the apparent heterogeneity regarding AMI-CFR in the group of tertiary level hospitals.

We further think to have gathered some evidence in favor of PCI over other treatments. Indeed, comparing our results with those of a previous study [12] and in line with a Swedish study, its practice seemed to have improved over time [12,59].

Finally, our analyses revealed important inter-hospital differences in medical practices but do not seem to indicate a systematic, early discharge practice of "patients about to die" in any hospital, intended to diminish its AMI-CFR. Also, the AMI-CFR of the hospital, admitting transferred out patients, seems to be protected to a certain extent by the modeling of shock, age group, gender and co-morbidity of this type of patients.

Nowadays, discharge records are used to compare AMI-CFRs between countries. It may well that these data suffer from similar limitations regarding the symptom-onset to needle-time, type of AMI, and that they are used both for reimbursement of the hospitals and for quality improvement purposes. Furthermore, in case of a hospital stay, taking place in more than one department, some countries collect discharge records of these partial stays, leading to an inflation of the denominator of the rate. As a consequence, one should very cautiously interpret differences in AMI-CFRs between countries. The OECD for instance suggests that this indicator should be considered in conjunction with length-of-stay and transfer rates and recommends risk adjustment for clinical factors [11].

### Quality improvement

In a perspective of quality improvement, implementation of evidence-based diagnostic and therapeutic practices and outcome monitoring have been shown to gradually improve outcomes in all types of hospitals and to decrease between-hospital variation [60,61]. More specifically, the measuring and tracking of performance is considered relevant to physicians, hospital managers, scientific bodies and policy-makers [62] and fit in the shift of focus, observed in recent years, from the "no blame" paradigm [63] to a more aggressive approach to poorly performing caregivers [64].

Our approach consisted mainly in a cautious ordering of findings in degrees of evidence by examining a hospital's departure both over the whole time period and over time in a perspective of improvement. Conscious of the data limitations, we refer to "screening" rather than "assessing" quality of care [65]. Therefore we avoided to establish a ranking of the hospitals, in itself a dubious technique [66], and to make our results available to the public by so-called report cards. The latter not only have been shown not to significantly improve composite process-of-care indicators for AMI [67] but also to lead to a rising post-discharge mortality rate, conceivably due to discharging patients in unstable conditions [68]. Further, taking the organizational nature of adverse events into account, we provided an anonymous feedback to the clinicians, the hospital management, the Belgian College of Cardiologists and the policymakers. As an input for the College of Cardiologists, we aimed at identifying the few, biggest outliers, which constitute an operationally manageable group to scrutinize with respect to the putative superior or substandard quality of care provided, and to propose corrective measures if necessary. Not outlying hospitals, displaying substantial departures, are suggested to proceed to an internal audit. Conversely, in our opinion it is more efficient to advise the vast majority of hospitals, finding themselves in the inconclusive zone, to comply with updated evidence-based guidelines.

To conclude, we are of the opinion that administrative data may provide hospitals and policy makers with enough evidence to encourage quality improvement initiatives. However, to measure progress it will be necessary to (1) routinely assess and assure the completeness and accuracy of the data; (2) to have univocal case definitions; and (3) to be able to trace patients across hospitals.

## Competing interests

The authors declare that they have no competing interests.

## Authors' contributions

WA conceived of the study, drafted the manuscript, and participated in the design of the study and in the statistical analysis. NT was responsible for the data management and participated in the statistical analysis. GM supervised the statistical analysis and participated in the design of the study. GDB participated in the design of the study (comparison MONICA). CV participated in the design of the study (cardiological aspects). MvS participated in the design of the study (epidemiological aspects).

All authors made important contributions to the interpretation of data and the redaction of the final manuscript, and approved it.

## Pre-publication history

The pre-publication history for this paper can be accessed here:

http://www.biomedcentral.com/1472-6963/10/334/prepub

## Supplementary Material

Additional file 1 **Charlson Comorbidity Index**. Click here for file

Additional file 2 **MONICA definitions**. Click here for file

Additional file 3 **Decision tree**. Click here for file
