# Supplementary material for: Do inter-hospital comparisons of in-hospital, acute myocardial infarction case-fatality rates serve the purpose of fostering quality improvement? An evaluative study
Source: BMC Health Serv Res. 2010 Dec 8;10:334. doi: 10.1186/1472-6963-10-334 (PMC3016357; doi:10.1186/1472-6963-10-334)
Supplement: Additional file 1 — Charlson Comorbidity Index. [file 1472-6963-10-334-S1.DOC]

MONICA diagnostic criteria

We reproduce here the MONICA diagnostic criteria^1^ as defined in the referenced article, free accessible at <http://circ.ahajournals.org/cgi/reprint/90/1/583> (accessed 08/19/2009) .

“Nonfatal events were classified as "definite" (NFl), "possible" (NF2), "ischemic cardiac arrest" (NF3), or "no myocardial infarction" (NF4). The major categories came from the European registers in the 1970s but were redefined quantitatively incorporating US criteria, in particular, the Minnesota code.

For a nonfatal event to be definite, there had to be either (1) progression of Minnesota codes on serial ECGs, ie, (a) progression from no Q wave to a definite Q wave; or (b) a lesser Q wave progression combined with progressive ST-segment depression, developing ST-segment elevation, or progressive T-wave inversion; or (c) persistent ST-segment elevation with progressive T-wave inversion in sequential daily ECGs or (2) cardiac enzyme levels twice the limit of normal, either with typical symptoms and an ECG that was not normal, or with an ECG progression labeled "probable" and lesser symptoms. Cases were placed in the nonfatal "possible" category of coronary events with typical prolonged (20 minutes) chest pain but lesser or no ECG and enzyme findings.

Fatal events were classified as "definite" (F1), "possible" (F2), "unclassifiable" (F9) (called "insufficient data" in the manual), or "no myocardial infarction or coronary death" (F4). Events were definite if they satisfied nonfatal definite criteria or if autopsy showed recent myocardial infarction or coronary thrombosis; possible coronary death involved suggestive

terminal symptoms or a CHD history in the absence of an autopsy or autopsy findings of chronic occlusive CHD or old infarction but no other pathological finding suggestive of a

fatal disease. No myocardial infarction or coronary death was allocated where another cause of death was implied by the clinical history or at autopsy.”

Reference List

(1) Tunstall-Pedoe H, Kuulasmaa K, Amouyel P, Arveiler D, Rajakangas AM, Pajak A. Myocardial infarction and coronary deaths in the World Health Organization MONICA Project. Registration procedures, event rates, and case-fatality rates in 38 populations from 21 countries in four continents. Circulation 1994; 90(1):583-612.
