# Supplementary material for: Do inter-hospital comparisons of in-hospital, acute myocardial infarction case-fatality rates serve the purpose of fostering quality improvement? An evaluative study
Source: BMC Health Serv Res. 2010 Dec 8;10:334. doi: 10.1186/1472-6963-10-334 (PMC3016357; doi:10.1186/1472-6963-10-334)
Supplement: Additional file 2 — MONICA definitions. [file 1472-6963-10-334-S2.DOC]

Scoring the co-morbidity index from secondary diagnoses by the Carlson’s co-morbidity index, D’Hoore implementation^26^ (CCI).

| Weights | Conditions | ICD-9 codes |
| --- | --- | --- |
| 1 | Myocardial infarct | 410, 411 |
|  | Congestive heart failure | 398, 402, 428 |
|  | Periferal vascular disease | 440 – 447 |
|  | Dementia | 290, 291, 294 |
|  | Cerebro-vascular disease | 430 – 433, 435 |
|  | Chronic pulmonary disease | 491 – 493 |
|  | Connective tissue disease | 710, 714, 725 |
|  | Ulcer disease | 531 – 534 |
|  | Mild liver disease | 571, 573 |
| 2 | Hemiplegia | 342, 434, 436, 437 |
|  | Moderate or severe renal disease | 403, 404, 580 - 586 |
|  | Diabetes | 250 |
|  | Any tumor | 140-195 |
|  | Leukaemia | 204 - 208 |
|  | Lymphoma | 200, 202, 203 |
| 3 | Moderate or severe liver disease | 070, 570, 572 |
| 6 | Metastatic solid tumor | 196 – 199 |
